# A Randomized Controlled Trial of the Effects of a Web-Based Intervention on Perceived Stress and Diet Quality Among First-Year University Students

**DOI:** 10.1089/tmr.2023.0041

**Published:** 2023-10-26

**Authors:** Joao F. Mota, Lorena C.C. Lopes, Claire F. Trottier, Steve T. Johnson, Jessica Lieffers, Carla M. Prado

**Affiliations:** ^1^ School of Nutrition, Federal University of Goiás, Goiânia, Brazil.; ^2^ Department of Bioscience, Centro Universitário de Mineiros–Unifimes, Mineiros, Brazil.; ^3^ Human Nutrition Research Unit, Department of Agricultural, Food and Nutritional Science, University of Alberta, Edmonton, Canada.; ^4^ Faculty of Health Disciplines, Athabasca University, Athabasca, Canada.; ^5^ College of Pharmacy and Nutrition, University of Saskatchewan, Saskatoon, Canada.

**Keywords:** internet-based intervention, wellness programs, mindfulness, stress

## Abstract

**Background:**

e-Health interventions can potentially improve health care. My Viva Plan^®^ (MVP) is a web-based program that focuses on mindfulness, nutrition, and physical fitness. The aim of this study was to evaluate the effects of this platform on stress indicators and diet quality among first-year university students.

**Methods:**

Ninety-seven university students were enrolled in a randomized, controlled clinical trial. Participants were randomized into control (*n* = 49) and MVP (*n* = 48) groups. Perceived stress was measured using the self-report Stress Indicator Questionnaire. Diet quality was assessed by the nutrient-rich foods index, and body composition was assessed by a hand-to-foot, multifrequency, bioelectrical impedance analysis.

**Results:**

There were no differences in physical, sleep, behavioral, emotional, and personal habit indicators between groups. Diet quality and body composition were similar between groups, except among women in the MVP group with decreased body fat (−1.2 ± 2.6 kg, *p* < 0.05). Participant engagement was low: 50% of the MVP group did not access the platform.

**Conclusions:**

The MVP web-based intervention was not associated with improvements in stress indicators, diet quality, and body composition, likely due to the characteristics of our cohort of healthy young individuals. Future studies should focus on enhancing motivational approaches to explore the potential of e-health interventions that improve health behavior.

Clinical Trial Registration number: NCT03579264A.

## Introduction

Adjusting to college is a major milestone in the life of young adults when emotional and mental disorders are common.^1^ As such, interventions to improve well-being in higher education students have emerged and have become even more relevant with the coronavirus disease 2019 (COVID-19) pandemic.^2^

Several e-health interventions have been used as an attempt to improve general health status,^3–5^ including behavioral interventions among university students. Self-reported physical activity has been inversely related to mental health problems in this population.^6^ Poor diet quality has also been associated with anxiety and depression.^7,8^ As one in three university students develop mental health problems over a 1-year period,^9^ behavioral interventions can play an essential role in optimizing health, including mental health.

e-Health interventions are a modern approach to improve health and are popular among young adults.^3,5^ Several clinical trials have investigated e-health as a tool to improve health behaviors, such as reducing anxiety, increasing physical activity, and improving diet quality.^7–10^ Although a wide variety of e-health applications have been developed, research on the effectiveness of these interventions is limited. The impact of using electronic interventions for behavioral change is controversial,^4,10,11^ and evidence suggests that only a quarter of app-based studies lead to significant health improvements compared with controls.^10^

Considering the myriad of factors that influence health choices and behavioral changes, multimodal interventions may offer an advantage for health management in general, which is the principle of the My Viva Plan^®^ (MVP, Revive Wellness, Inc., Edmonton, AB, Canada). The MVP, which is not available on the App Store or Google Play Store, is a web-based program (www.myvivainc.com) with associated costs and mobile-responsive features, similar to an app.

This program emphasizes three pillars of health—mindfulness, nutrition, and physical fitness—and uses a self-guided approach to monitor health behaviors, including food intake, physical activity, and perceived stress. As such, this technology may be attractive to university students seeking to optimize their health. Thus, the aim of this randomized clinical trial was to evaluate the effects of a web-based mindfulness, nutrition, and physical activity platform on stress indicators and diet quality among first-year university students.

## Materials and Methods

### Research design and participants

This was a 12-week, randomized, controlled clinical trial registered at ClinicalTrials.gov and a detailed study protocol has been previously published.^4,12^ Screenshots of select platform interfaces are available in our previous publications.^4,12^

This study was conducted according to the guidelines outlined in the Declaration of Helsinki and approved by the University of Alberta Ethics Board (Pro00079680) and complied with the standards set out in the Canadian Tri-Council Policy statement on the use of human participants in research. Participants were recruited during the fall and winter terms (September 2018 to December 2018 and January 2019 to April 2019, respectively).

Inclusion criteria were males and females, 17 to 30 years of age, who were able to complete all study assessments in their first year of undergraduate studies. Exclusion criteria included any self-reported disordered eating; self-reported untreated depression, anxiety, or mood disorder; pregnancy or lactation; not having a device to access the internet (i.e., computer, smartphone, or tablet); not able to communicate in English; or having an electronic implantable device (due to body composition assessment).

Participants were assumed to have strong computer and internet literacy. Participants received $50 (CAD) cash and 1 year of free membership to MVP after completing all study assessments.

Using block randomization by sex, participants were assigned to the control group (no intervention) or the intervention group (MVP use); block sizes randomly alternated between blocks of two and four to minimize the risk of predictable group allocation. An independent biostatistician performed the allocation sequence and uploaded into Research Electronic Data Capture (REDCap).

Blinding was not possible as available staff needed to provide access to the platform and monitor its use and conducted qualitative interviews previously published.^12^ Participants allocated to the intervention group received access to the MVP for a 12-week period. The intervention was exclusively automated, with no human interaction.

The control group did not receive access to MVP and were asked to continue their daily habits.

### Additional platform details

MVP is guided by the social cognitive and self-regulation theory^13^ and encompasses three core components: “My Mind,” “My Nutrition,” and “My Fitness.” The “My Mind” component encouraged participants to set goals and reflect daily on stress, diet, exercise, and progress on their goals. The “My Nutrition” component provided participants the option to customize meal plans based on their needs and preferences as well as create grocery lists. This component also provided users access to recipes and suggestions for healthy choices when eating at restaurants. In the “My Fitness” component, participants had access to video tutorials for exercises and a fitness scheduler.

Participants were asked to use this platform as frequently as possible and their engagement was assessed by the number of logins over the course of the intervention. A video tutorial within the MVP provided guidance on the platform's use, which is described elsewhere.^12^ No additional training on MVP was provided by researchers. Those in the control group were asked to maintain their usual lifestyle throughout the study and did not receive access to MVP during the study.

### Perceived stress

Perceived stress was measured using the self-reported Stress Indicator Questionnaire (SIQ) at baseline and after 6 and 12 weeks.^13^ This SIQ is divided into five indicator categories, including physical (*n* = 21 items), sleep (*n* = 5 items), behavioral (*n* = 17 items), emotional (*n* = 20 items), and personal habits (*n* = 9 items).

Possible responses for items in each indicator category were measured using a five-point Likert scale: 1—*never*, 2—*almost never* (less than 2 h a week), 3—*some of the time* (one and one-half days a week), 4—*most of the time* (3 days a week), and 5—*almost always* (5 days a week). Total scores range from 73 to 365 points, with higher total scores indicating higher levels of perceived stress.

### Diet quality

The nutrient-rich foods index, version 9.3,^14,15^ a formal scoring system to assess the nutrient density of individual foods, was used to assess diet quality. The diet quality was based on the sum of the percentage of daily values for nine nutrients (protein, dietary fiber, vitamin A, vitamin C, vitamin D, calcium, iron, potassium, and magnesium) minus the sum of the percentage of maximum recommended values for three nutrients to limit (added sugars, saturated fatty acids, and sodium), with all daily values calculated per 100 kcal and capped at 100% to limit the effect of fortified foods. Vitamin E was replaced with vitamin D due to prevalence of vitamin D deficiency, which is considered a public health problem.^4,16^

### Anthropometry and body composition assessment

Anthropometric and body composition measurements were obtained at baseline and after 12 weeks. Trained staff asked participants to wear light clothing and remove their footwear for these assessments. Body weight was measured using a calibrated digital scale (Health o meter^®^ Professional Remote Display, Sunbeam Products, Inc., FL) to the nearest 0.1 kg. Height was measured using a Heightronic Digital Stadiometer (QuickMedical, Issaquah, WA) to the nearest 0.1 cm. Body–mass index was defined and evaluated using previously defined categories.^17^

Body composition was assessed by a hand-to-foot, multifrequency, bioelectrical impedance analysis using the QuadScan 4000^®^ (BodyStat, Isle of Wight, United Kingdom). Participants were instructed to fast for 5 h (drinking water only) and avoid intense physical activity for 12 h before testing. Before testing, participants were instructed to remove their shoes, socks, and all metal accessories before lying in a supine position. Fat mass, lean mass, and total body water were estimated based on device-specific equations.

### Statistical analyses

The primary study outcome was the change in perceived stress between groups, while the secondary outcome was change in diet quality. All other measurements were exploratory. It was hypothesized that the use of MVP would reduce perceived stress and improve diet quality among first-year university students. With an anticipated attrition rate of 20%, we aimed to recruit 100 participants. A sample size of 33 participants was estimated (power1-β = 0.95) based on reduction in perceived stress scale scores of distressed adults after an internet-based stress management intervention (effect size = 0.74).^18^ G*Power^®^ software was used for sample size estimation (version 3.1.9.2; Heinrich-Heine-Universität Düsseldorf, Düsseldorf, Germany).^19^

Data distribution was evaluated using the Kolmogorov–Smirnov test. Homogeneity of variance was verified with Levene's test. Results are expressed as mean ± standard deviation. A paired *t*-test was employed to assess within-group differences. Differences between groups were tested using per-protocol and intention-to-treat analyses.

In the per-protocol analysis, a 2 × 3 (group [intervention, control] × time [baseline and 6 and/or 12 weeks]) analysis of covariance was used, with baseline scores entered as covariates. When a significant interaction was found, Duncan's method for multiple comparisons was used.^20^ For the intention-to-treat analysis, all participants who were randomized and had baseline information were included and analyzed according to original treatment assignment using generalized estimating equations, unstructured correlation, and maximum likelihood estimation.

The model (linear or gamma) was chosen based on the model fit quality Quasilikelihood under the independence model criterion (QIC). When necessary, the least significant difference *post hoc* test was used. All values are described as means and Wald 95% confidence intervals. The diet quality index score was tested using analysis of variance (ANOVA 2x2). All analyses were performed using SPSS software, version 25.0 (IBM Corp., Armonk, NY), with *p* ≤ 0.05 considered significant.

## Results

**FIG. 1.**
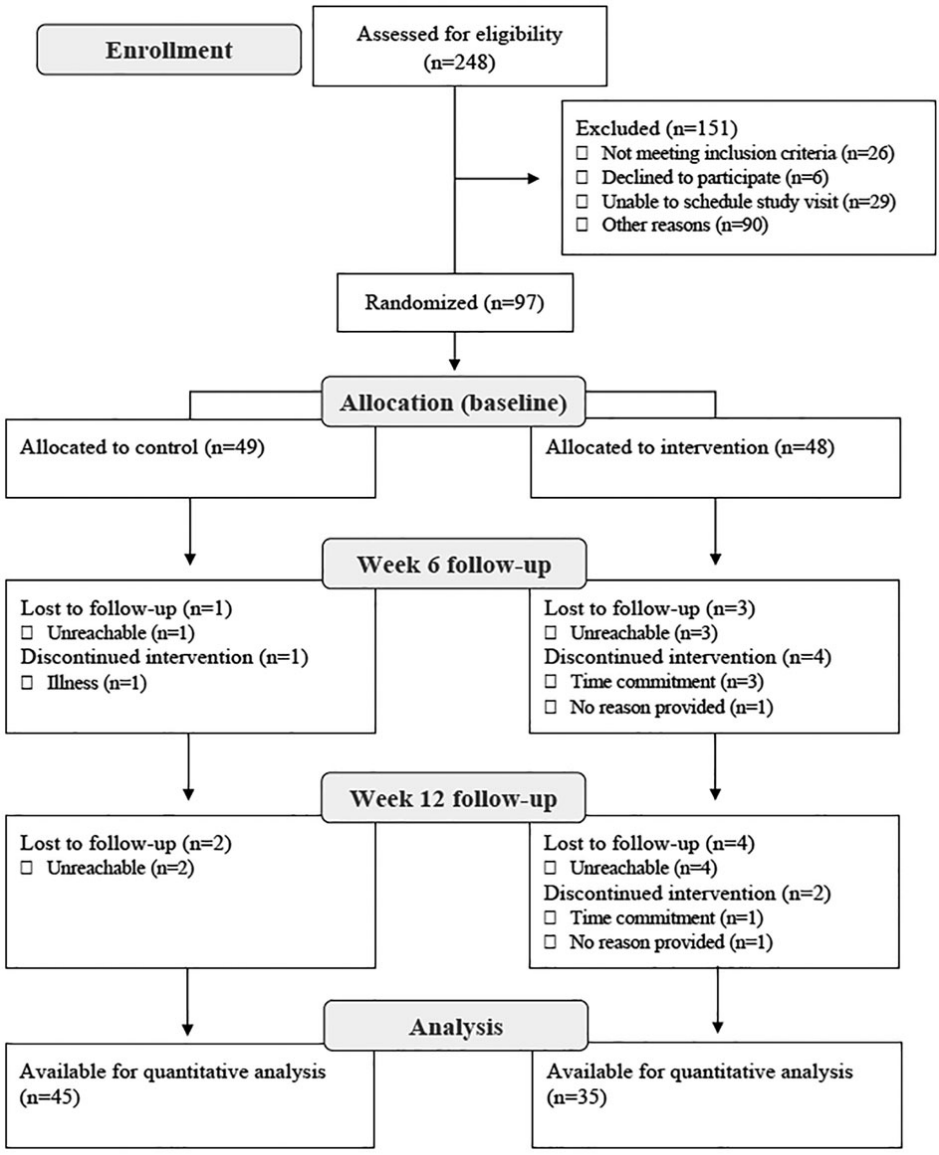
Study flowchart.

A total of 248 undergraduate students were assessed for eligibility (Fig. 1); 145 did not meet the inclusion criteria and 6 declined to participate. Therefore, 97 participants were randomized into the control (*n* = 49) and MVP (*n* = 48) groups. Table 1 presents the baseline characteristics of the randomized sample. For the per-protocol analysis (*n* = 80), 10 participants dropped out of the study because they were lost to follow-up (*n* = 3 in the control group and *n* = 7 in the intervention group) and 7 discontinued the intervention (*n* = 1 in the control group and *n* = 6 in the intervention group).

**Table 1.** Baseline Characteristics of Participants

	Control group (***n*** = 49)				MVP group (***n*** = 48)			
	Women (***n*** = 35)		Men (***n*** = 14)		Women (***n*** = 34)		Men (***n*** = 14)	
	Mean	SD	Mean	SD	Mean	SD	Mean	SD
Age (years)	18	1	19	3	18	2	18	1
Weight (kg)	60.9	9.1	73.3	13.5	61.8	8.7	73.1	12.6
BMI (kg/m^2^)	22.8	2.9	23.2	5.4	23.2	3.1	23.7	3.6
Body fat (%)	26.6	4.4	15.5	3.7	27.2	6.1	16.9	6.7
Body fat (kg)	16.3	4.4	12.0	8.5	17.3	5.4	12.8	6.6
Lean mass (kg)	44.5	6.0	61.1	8.1	45.1	4.6	60.4	8.5

Stress indicators								
Physical	44.5	6.0	41.8	6.5	45.6	9.1	41.7	8.0
Sleep	10.9	3.9	10.1	2.9	11.3	3.9	10.7	1.6
Behavioral	33.6	4.9	31.2	3.4	31.3	5.2	31.6	4.2
Emotional	48.0	11.5	43.5	8.1	45.3	12.2	47.8	10.4
Personal habits	28.2	4.6	26.1	3.9	26.1	5.3	24.8	5.5

BMI, body–mass index; MVP, My Viva Plan; SD, standard deviation.

The effects of the intervention on stress indicators are shown in Table 2. There were no differences in physical, sleep, behavioral, emotional, and personal habit indicators. These results remained similar when the intention-to-treat analysis was performed (Table 2).

**Table 2.** Effects of My Viva Plan on Stress Indicators

***Per protocol*** ^*^	Control group						MVP group						***Time × group interaction***
	Baseline		Six weeks		Twelve weeks		Baseline		Six weeks		Twelve weeks		
	**Mean**	**SD**	**Mean**	**SD**	**Mean**	**SD**	**Mean**	**SD**	**Mean**	**SD**	**Mean**	**SD**	***p*** ^**a**^
Physical	44.3	7.9	46.7	10.2	44.8	9.3	45.7	8.7	46.6	10.7	46.5	11.3	0.55
Sleep	10.5	3.1	11.2	3.1	10.3	3.1	11.4	3.2	11.2	3.6	10.9	3.1	0.39
Behavioral	32.7	4.3	34.0	5.9	33.5	4.9	31.5	4.6	32.3	6.9	31.9	6.9	0.90
Emotional	46.8	10.7	51.2	12.5	48.6	13.3	47.2	1.08	49.6	13.1	49.2	13.5	0.41
Personal habits	28.1	4.2	27.8	5.5	27.4	5.2	26.3	4.8	26.1	5.2	26.2	5.5	0.80

***Intention to treat*** ^**^	Mean	95% CI	Mean	95% CI	Mean	95% CI	Mean	95% CI	Mean	95% CI	Mean	95% CI	***p*** ^b^
Physical	44.5	42.4–46.7	47.1	44.3–50.0	45.2	42.6–47.8	44.5	42.0–47.0	46.0	43.1–49.1	45.9	42.6–49.4	0.94
Sleep	10.7	9.6–11.6	11.3	10.5–12.2	10.4	9.6–11.4	11.2	10.3–12.1	11.2	10.2–12.3	10.8	9.9–11.8	0.73
Behavioral	32.9	31.7–34.2	34.1	32.5–35.8	33.6	32.2–35.0	31.4	30.1–32.7	31.7	29.7–33.8	31.5	29.4–33.7	0.25
Emotional	46.7	43.9–49.9	51.2	47.8–54.8	48.6	44.9–52.5	45.9	42.8–49.1	49.1	45.2–53.3	48.6	44.6–53.1	0.73
Personal habits	27.6	26.4–28.8	27.3	25.8–28.9	27.0	25.5–28.6	25.7	24.3–27.2	25.4	23.9–26.9	25.7	24.0–27.4	0.70

^*^ *n* = 45 (control group) and *n* = 35 (MVP group).

^**^ *n* = 49 (control group) and *n* = 48 (MVP group).

^a^ ANCOVA with adjustment for the baseline observation when variables were not homoscedastic at baseline in the per-protocol analysis or ^b^intention-to-treat analysis adjusted by sex.

ANCOVA, analysis of covariance; CI, confidence interval.

There was no effect of the MVP intervention on body composition and diet quality (Table 3). In the additional analysis considering sex, body fat decreased after the intervention in women only in the MVP group (−1.2 ± 2.6 kg, *p* < 0.05, Supplementary Table S1), however, without differences between groups. Participant engagement was low; overall, 50% of the MVP group did not access the platform. Among those who accessed the platform, 37.5% accessed it less than 10 times, 29.2% accessed it between 10 and 24 times, and 33% accessed it more than 24 times.

**Table 3.** Effects of My Viva Plan on Body Composition and Diet Quality

	Control group				MVP group				***Time × group interaction***
	Baseline		Twelve weeks		Baseline		Twelve weeks		
***Per protocol*** ^*^	Mean	SD	Mean	SD	Mean	SD	Mean	SD	***p*** ^a^
Weight (kg)	64.2	12.1	65.4	11.3	65.4	11.2	65.6	11.6	0.84
BMI (kg/m^2^)	22.7	3.8	23.6	3.2	22.8	3.7	23.1	5.1	0.27
Body fat (%)	22.9	7.7	21.9	8.2	25.4	6.4	23.5	6.7^d^	0.22
Body fat (kg)	14.7	6.1	14.2	6.3	16.9	5.3	15.5	5.7^d^	0.23
Lean mass (kg)	49.4	10.4	50.4	10.6^c^	49.1	8.8	50.1	9.7^c^	0.96
Diet quality score	53.6	24.9	54.7	25.9	53.7	21.7	45.9	20.2^c^	0.11

***Intention to trea***t^**^	Mean	95% CI	Mean	95% CI	Mean	95% CI	Mean	95% CI	***p*** ^b^
Weight (kg)	64.2	61.0–67.7	64.7	64.2–70.5	65.1	62.0–68.6	65.3	62.5–68.0	0.42
BMI (kg/m^2^)	22.9	21.0–23.9	23.0	22.0–26.0	23.3	22.4–24.2	23.4	22.5–24.4	0.71
Body fat (%)	23.4	21.4–25.6	22.3	20.1–24.7	24.2	22.1–26.4	22.4	16.5–22.8	0.70
Body fat (kg)	15.7	13.6–18.0	14.6	12.6–16.9	14.9	12.9–17.1	13.5	20.8–25.9	0.52
Lean mass (kg)	49.2	46.2–52.1	50.6	47.0–47.8	49.6	47.0–52.0	50.6	47.8–53.5	0.42
Diet quality score	53.7	50.4–56.0	54.4	49.5–57.5	53.4	51.4–56.3	45.9	47.3–51.2	0.67

^*^ *n* = 45 (control group) and *n* = 35 (My Viva group).

^**^ *n* = 49 (control group) and *n* = 48 (My Viva group).

^a^ ANCOVA with adjustment for the baseline observation when variables were not homoscedastic at baseline in the per-protocol analysis or ^b^intention-to-treat analysis adjusted by sex.

^c^ *p* < 0.05 and ^d^*p* < 0.01 versus baseline by paired *t*-test.

## Discussion

This study examined the effects of a web-based tool on perceived stress, diet quality, and body composition in first-year university students. No significant changes were observed in any outcome measure. Although body fat mass decreased in women in the intervention arm, there were no differences between the intervention and control groups. These findings should be interpreted with caution in view of the very low access of the MVP platform.

Our findings are consistent with previous randomized clinical trials involving e-health, in which college students showed low adherence to the intervention.^21^ A previous study highlighting difficulties in maintaining user engagement showed that access to a popular platform (Localytics) across more than 2.7 billion devices and 37,000 mobile and web apps was low; 23% of users used the app only once, while 39% of users returned to the app for 11 or more sessions.^22^

Considering these low engagement rates, constant follow-up and encouragement may be needed to improve adherence in future studies involving university students. A limitation of this study was the lack of control over usage time when accessing the program.

The low rate of access hereby observed may be related to the characteristics of our cohort of healthy young individuals. Previous studies in clinical populations, such as individuals with cancer,^23^ hypertension,^24^ or asthma,^25^ have reported higher engagement and adherence to e-health interventions compared with ours. These findings suggest that the need for disease self-monitoring and care may play a special role in motivation and adherence to web-based interventions.

We speculate that health awareness and motivation for disease prevention are lower in this group of healthy and young individuals. Notably, we did not assess readiness to change and therefore we are unable to comment on whether students were interested in engaging in preventive health behavior or if their motivation was related to information access (e.g., body composition data) and/or to the study compensation.

An additional consideration is that user engagement is shaped by socio-contextual influences, such as the role played by family members and the broader cultural setting.^26^ In fact, we have previously shown that living arrangements, financial conditions, and high stress levels during examinations appeared to be important barriers to engaging with the MVP application.^10^ Thus, maintaining engagement can be especially difficult for university students, and low adherence, as reported previously,^27,28^ could explain our findings.

Importantly, we did not provide health care professional support or systematic reminders/encouragement for participants to access MVP, which may influence the adherence rate. Therefore, we suggest that the combined use of the e-health tool and other interventions such as group sessions, coaching calls, text messages, e-mails, and face-to-face contact may improve engagement among university students.^11,29^

Findings from studies exploring e-health tools also suggest improved effectiveness in a shorter term (between 4 and 8 weeks), which is likely due to decreases in adherence and motivation.^4,5^ In a systematic review of technology-driven behavior change techniques, researchers found that 63% of e-health-based interventions were effective in the short term (<3 months), whereas only 33% were effective with long-term use (≥12 months).^30^

In our study, the first follow-up assessment occurred at 6 weeks (stress indicators); however, no treatment effect was found. In view of the expected decline in engagement associated with the use of web-based tools, concurrent health care professional support is therefore recommended to improve engagement with the intervention.^4^ Although MVP provides this option for subscribers, it was not used in the current study.

Interestingly, our previous qualitative analysis indicated that participants perceived an increase in awareness regarding all three MVP educational pillars (i.e., mindfulness, nutrition, and fitness).^4^ However, this was not enough to reflect platform access and consequently the low rate of access to MVP and its potential impact on stress indicators.

Future studies should explore how to engage and motivate participation of this population group of young healthy individuals. In an open-ended and optional text box within the Mobile Application Rating Scale,^31^ 18 of 35 participants noted common barriers to platform use: being busy with their academics (*n* = 3 of 18); accessibility concerns/preference for a smartphone-based app (*n* = 6 of 18); and living arrangements on residence that limit usefulness of the platform (*n* = 1 of 18).^4^

Finally, our intervention started at the beginning of the academic year/semester for first-year university students. This may also have affected stress and anxiety markers, which are lower during this period compared with the examination period.^32^ This is nonetheless arguable as students may also experience stress when starting university. This remains to be tested in future studies.

In conclusion, the MVP web-based intervention was not associated with improvements in stress indicators, diet quality, and body composition, which may be explained by the limited platform access by participants. Given the challenges associated with engagement, motivational approaches—including, but not limited to, health care professional support—should be included to fully explore the potential of this platform to improve the health behavior of first-year university students.

## Supplementary Material

Supplemental data

## Abbreviations Used

ANOVA: analysis of variance
ANCOVA: analysis of covariance
CI: confidence interval
COVID-19: coronavirus disease 2019
MVP: My Viva Plan
REDCap: Research Electronic Data Capture
SD: standard deviation
SIQ: Stress Indicator Questionnaire
