# Supplementary material for: A Randomized Controlled Trial of the Effects of a Web-Based Intervention on Perceived Stress and Diet Quality Among First-Year University Students
Source: Telemed Rep. 2023 Oct 26;4(1):327–35. doi: 10.1089/tmr.2023.0041 (PMC10615051; doi:10.1089/tmr.2023.0041)
Supplement: Supplemental data [file Suppl_TableS1.docx]

**Supplementary material**

***A randomized controlled trial of the effects of a web-based intervention on perceived stress and diet quality among first-year university students.***

**Table 1. Effects of My Viva Plan^®^ on body composition between sexes.**

|  | Control group  (Men, N=14) | | | | | My Viva Plan group  (Men, N=9) | | | | | Control group  (Women, N=31) | | | | | | My Viva Plan group  (Women, N=26) | | | | | | | *Time x Group* |
| --- | --- | --- | --- | --- | --- | --- | --- | --- | --- | --- | --- | --- | --- | --- | --- | --- | --- | --- | --- | --- | --- | --- | --- | --- |
|  | Baseline | | 12-week | |  | Baseline | | 12-week | |  | Baseline | | 12-week | |  | Baseline | | | 12-week | | |  | |  |
|  | mean | SD | mean | SD | *p ^a^* | mean | SD | mean | SD | *p ^a^* | mean | SD | mean | SD | *p ^a^* | mean | | SD | | mean | SD | | *p ^a^* | *p ^b^* |
| Weight (kg) | 73.3 | 13.5 | 73.8 | 12.8 | 0.42 | 75.7 | 13.7 | 76.8 | 13.0 | 0.56 | 60.0 | 8.9 | 60.3 | 8.8 | 0.66 | 61.8 | | 7.8 | | 61.7 | 8.2 | | 0.81 | 0.64 |
| BMI (kg/m^2^) | 23.2 | 5.4 | 23.3 | 5.1 | 0.46 | 24.2 | 4.2 | 22.5 | 8.8 | 0.30 | 22.5 | 2.8 | 22.6 | 2.9 | 0.63 | 23.4 | | 2.7 | | 23.4 | 2.9 | | 0.88 | 0.64 |
| Body Fat (%) | 15.5 | 8.3 | 13.1 | 7.1 | 0.18 | 20.1 | 6.2 | 17.1 | 8.2 | 0.86 | 26.2 | 4.4 | 25.9 | 4.9 | 0.51 | 27.3 | | 5.5 | | 25.7 | 5.3 | | 0.01 | 0.13 |
| Body Fat (kg) | 12.0 | 8.5 | 10.4 | 7.8 | 0.11 | 15.6 | 6.5 | 13.7 | 7.9 | 0.96 | 15.9 | 4.3 | 15.9 | 4.8 | 0.96 | 17.3 | | 4.9 | | 16.1 | 4.8 | | 0.02 | 0.08 |
| Lean Mass (kg) | 61.1 | 8.1 | 63.4 | 6.8 | 0.02 | 60.1 | 8.1 | 63.1 | 8.3 | 0.79 | 44.1 | 6.1 | 44.5 | 5.5 | 0.35 | 45.3 | | 4.5 | | 45.7 | 5.0 | | 0.45 | 0.90 |

^a^ Paired t-test and ^b^ ANCOVA with adjustment for the baseline observation when variables were not homoscedastic.
